# Supramolecular PDDA/PEDOT:PSS Biosensor for Early Pancreatic Cancer Detection via CA19-9: Clinical Validation on Human Blood Samples

**DOI:** 10.1021/acsomega.5c11381

**Published:** 2026-01-22

**Authors:** Gabriella Onila N. Soares, Andrey C. Soares, Ronaldo Dias, Rafael Kemp, Débora Gonçalves

**Affiliations:** † Materials Engineering Department, Engineering School, 28133 University of São Paulo, São Carlos, São Paulo 13563-120, Brazil; ‡ Department of Physics, Federal University of Amazonas, Manaus, Amazonas 69067-005, Brazil; § Ribeirão Preto Medical School, University of São Paulo, Ribeirão Preto, São Paulo 14040-900, Brazil; ∥ Institute of Physics of São Carlos, University of São Paulo, São Carlos, São Paulo 13566-590, Brazil

## Abstract

Pancreatic cancer has one of the highest mortality rates, and early detection remains a challenge, significantly limiting therapeutic strategies. In this study, we present the clinical validation of a novel multilayered capacitance-based biosensor for early pancreatic cancer detection. Poly­(diallyldimethylammonium chloride) and (poly­(3,4-ethylenedioxythiophene):polystyrenesulfonate) (PDDA/PEDOT:SS) were physically adsorbed onto gold interdigitated electrodes via self-assembly, followed by surface functionalization with CA19-9 antibodies. Upon selective binding of the CA19-9 biomarker, the adsorption kinetics indicated that the system reached equilibrium within 7 min. Polarization modulation infrared reflection absorption spectroscopy, atomic force microscopy analysis, and electrical measurements confirmed the successful functionalization of the biosensor surface. The interaction between CA19-9 and the functionalized surface was evaluated using electrical impedance spectroscopy. The calibration curve was best fitted to the Langmuir–Freundlich model, and all data sets were processed by visual analysis (IDMAP). Key characteristics of the devices — sensitivity and selectivity — demonstrate a limit of detection of 0.01 U/mL, limit of quantification of 0.03 U/mL, and specificity toward CA19-9. Analyses were conducted on 24 blood samples collected from patients at different stages of the disease. The good performance at low and moderate CA19-9 concentrations was supported by IDMAP and Bland–Altman statistical analysis. The results confirmed the biosensor’s potential as an innovative, sensitive, and selective tool for early detection of pancreatic cancer, with the possibility of future technology transfer to the Brazilian Health System.

## Graphical abstract

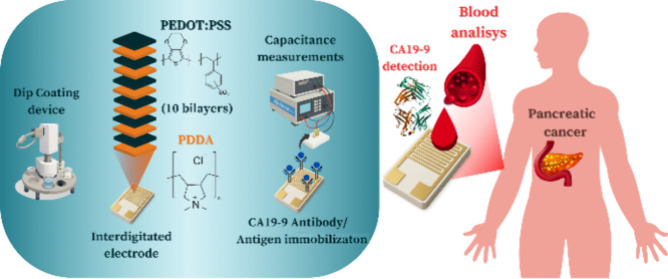

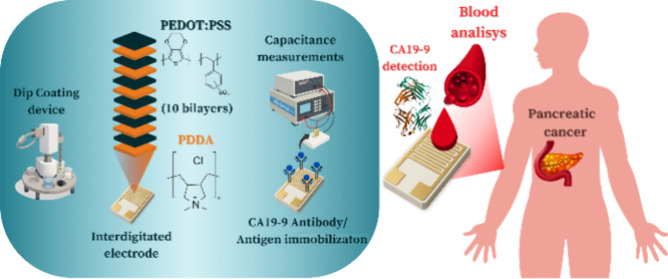

## 1 Introduction

Pancreatic cancer remains one of the most significant challenges in modern oncology due to its high mortality and late diagnosis, which limit treatment options and reduce survival rates.^1^ According to the National Cancer Institute,^2^ when diagnosed at an advanced stage (51% of diagnosed patients), the 5 year relative survival rate is only 3%. The lack of sensitive, selective, and practical early detection methods hampers effective treatment. Innovative, accurate, and noninvasive diagnostic approaches hold strong potential to improve outcomes and support public healthcare.^3^

Biosensors based on functional materials, particularly those analyzed using electrochemical impedance spectroscopy (EIS), offer new paths for early disease diagnosis.^4^ Beyond sensitivity and specificity, these devices offer portability, flexibility, and multifunctionality.^3^ Moreover, biosensors enable continuous and real-time monitoring of various biomarkers, including CA19-9, which are currently used to monitor patients with pancreatic cancer.^5^ Wang et al.^6^ developed an electrochemical biosensor for CA19-9 detection using gold nanoparticles biosynthesized from mint extract. The sensor showed a broad linear range (0.1–100 U/mL, *R* ^2^ = 0.998) and a low limit of detection (LOD) (0.05 U/mL). According to the authors, the biosensor outperformed ELISA in terms of speed and ease of use; however, a more detailed analysis is necessary to compare, in fact, the biosensor with the ELISA technique. Thapa et al.^7^ developed a biosensor by assembling polyethylenimine (PEI) and carbon nanotubes (CNTs) in a layer-by-layer thin film on gold interdigitated electrodes, followed by antibody immobilization via NHS/EDC chemistry. The device achieved a LOD of 0.35 U/mL for CA19-9 in buffer.

The adsorption behavior followed the Langmuir–Freundlich isotherm, and the sensor demonstrated high selectivity against common interferents. Despite significant advances in the development of biosensors for cancer detection, their large-scale application still faces several challenges, including instability, lack of standardization, relatively high production costs, and noisy signals. Although these devices have shown promising results under laboratory conditions, few have demonstrated robust commercial viability or undergone validation with real clinical samples from affected patients.^7–9^ Among well-known conjugated polymers, poly­(3,4-ethylenedioxythiophene):polystyrenesulfonate (PEDOT:PSS) stands out due to its mixed ionic–electronic conductivity, biocompatibility, and structural tunability. When combined with cationic poly­(diallyldimethylammonium chloride) (PDDA) in polyelectrolyte multilayer architectures by self-assembly by physical adsorption (Layer-by-Layer, LbL) deposition, these films exhibit improved capacitance response and stability, making them an attractive platform for biosensing.^10,11^

In this study, we report on the design, fabrication, and validation of multilayered PDDA/PEDOT:PSS biosensors functionalized with CA19-9 antibodies on gold interdigitated electrodes. This architecture has not been previously reported for CA19-9 detection or pancreatic cancer diagnostics. The devices were systematically characterized in terms of morphology, adsorption kinetics, sensitivity, selectivity, and detection mechanisms, and their analytical performance was benchmarked against ELISA using clinical blood samples. To improve the interpretability of high-dimensional EIS data, advanced visualization methods, such as Interactive Document Maps (IDMAP), were employed, allowing for the robust classification of biomarker concentrations and the quantification of selectivity using the silhouette coefficient. We also employed Bland-Altman statistical analysis to clinical validation of the biosensor on human blood samples. Our findings demonstrate that this architecture enables the rapid and selective detection of CA19-9, approaching ELISA sensitivity at low to moderate concentrations. Therefore, this research introduces a promising pathway toward diagnostic tools for pancreatic cancer, bridging the gap between advanced materials engineering and urgent clinical needs.

## 2 Experimental Procedure

### 2.1 Interdigitated Electrode Fabrication and Modification

Interdigitated electrodes (IDEs) (25 μm spacing) were fabricated via photolithography in BK7 glass substrates, which were coated with a chrome layer of 100 μm and a thin layer of gold (20 μm), using physical vapor deposition (PVD). The unexposed material was then removed by selective etching.^12^ Then, the IDEs were subjected to UV/Ozone and were functionalized by the LBL technique with poly­(diallyldimethylammonium chloride) (PDDA, Sigma-Aldrich) and (poly­(3,4-ethylenedioxythiophene):polystyrenesulfonate) (PEDOT:PSS, Clevios PH 1000, Heraeus), using a dip-coating system. The construction was based on assembling 10 bilayers, with each bilayer cycle consisting of: (i) 10 min of immersion in PDDA solution (10 g/L — Milli-Q water), (ii) 30 s immersion in Milli-Q water under 50 kHz agitation, (iii) drying the edges on paper, (iv) 10 min of immersion in PEDOT:PSS (6.5 g/L — Milli-Q water), and then (v) 30 s immersion in Milli-Q water under 50 kHz agitation, followed by (vi) drying the edges on paper. Then, the IDEs were dried on the hot plate at 70 °C for 5 min. Following film modification, the interdigital area was delimited using a waterproof adhesive vinyl. To evaluate the film buildup effects on the multilayer architecture electrical properties, capacitance measurements were carried out on PDDA/PEDOT:PSS films constructed with 2, 4, 8, and 10 bilayers, using an impedance analyzer (Solartron 1260A, Farnborough, England). The bilayer was fabricated using the same protocol described above (Layer-by-Layer), and the capacitance spectra were recorded over the full frequency range (1 to 10^6^ Hz). These measurements allowed assess the progressive increase in signal intensity as a function of film thickness and to determine the optimal number of bilayers for sensor fabrication. Then, CA19-9 antibodies (AB) (Abcam) and antigens (AG) (Abcam) were immobilized onto the surface via drop-casting through physical adsorption. For this, 10 μL of anti CA19-9 was placed on the interdigital surface for 1 h and, then washed with Milli-Q water. After 10 μL of AG CA19-9 (0.01, 0.1, 1, 5, 20, 37, 60, 100, and 300 U/mL) was placed on the interdigital surface for 10 min, it was then washed with Milli-Q water.

### 2.2 Morphology

Atomic force microscopy (AFM) was used to characterize the film morphology and confirm antibody immobilization, focusing on surface homogeneity and quantitative parameters such as RMS roughness. Measurements were performed using a BRUKER AFM system, operating with a 0.1 N force and a scanning rate of 0.6 Hz.

### 2.3 Adsorption Kinetics

Adsorption kinetics were evaluated by monitoring the electrical signal (capacitance variation) over time after the introduction of CA19-9 antigens onto the functionalized electrode surface. Measurements were carried out at 1 Hz using the Solartron analytical system (Solartron 1260A, Farnborough, England) for 20 min, with AC potential 50 mV and potential DV 0 mV. The data were fitted to pseudo-first-order, pseudo-second-order, and Elovich adsorption models to evaluate the mechanism of CA19-9 adsorption onto the PDDA/PEDOT:PSS multilayer films.

### 2.4 Calibration Curve

The calibration curve was constructed based on normalized capacitance (F*) data (eq 1) acquired at 1 Hz using the Solartron system. *F*_0_ is the capacitance signal of CA19-9 antibody, *F*_*x*_ is the capacitance signal of CA19-9 antigen at concentration *x* (U/mL). Aliquots of 10 μL of AG CA19-9 at different concentrations were deposited onto the surface of the interdigitated electrodes for 10 min. After immobilization, the electrodes were washed with Milli-Q to remove unbound antigen. The tests were conducted in triplicate using three independent sensors, and each capacitance measurement was also performed in triplicate to ensure reproducibility. Standard deviation (σ) analysis was performed according to eq 2, where *n* is the sample size and $\overset{®}{F^{*}\;}$ is the sample mean.

$$F^{*}=\frac{\left(F_{0}-F_{x}\right)}{F_{0}}$$ 1

*F*_0_ capacitance of AB and *F*_*x*_ capacitance of AB + AG (at concentration *x*)­

$$\sigma =\sqrt{\frac{\sum (F^{*}-\overset{®}{F^{*}}{)}^{2}}{n}}$$ 2

### 2.5 Sensitivity and Selectivity Tests

Limit of detection (LOD) and limit of quantification (LOQ) were measured following IUPAC standards (eqs 3 and 4):^13,14^

$$\mathrm{LOD}=3\times \sigma_{e}/\alpha$$ 3

LOQ=3,3×LOD 4

where: σ_e_ is the noise standard deviation and α slope of the calibration curve.

To minimize underestimation and overestimation caused by the curve’s nonlinearity, the data were plotted on a logarithmic scale, allowing a more accurate determination of the slope. The selectivity of the biosensors was assessed through two complementary approaches: (i) evaluating the response in the absence of immobilized antibodies (negative control), and (ii) analyzing the biosensor response to different unrelated biomarkers: P53 (1.0 mg/mL), PSA (1.0 mg/mL), SARS-CoV-2 (0.9 ng/mL) antigens was evaluated and compared to that of CA19-9 (1.0 U/mL) to assess the presence of interferers. The data was processed using the Interactive Document Map technique with the Projection Explorer software (IDMAP).

### 2.6 PM-IRRAS

Polarization Modulation Infrared Reflection–Absorption Spectroscopy (PM-IRRAS) measurements were performed using a KSV PMI 550 instrument (Helsinki, Finland) with a spectral resolution of 8 cm^–1^ over a total acquisition time of 600 s, with a gold spectrum as a reference. A solid gold substrate was placed beneath the IDEs to ensure optimal reflectivity. The analysis was conducted to characterize (i) the individual thin films of PDDA, PEDOT:PSS, and the multilayered PDDA/PEDOT:PSS architecture (10 bilayers), and (ii) the adsorption behavior of CA19-9 antibodies/antigens onto the film.

### 2.7 Blood Sample Analysis

The Ribeirão Preto Medicine School of the University of São Paulo provided a total of 24 human blood samples. Among them, 6 samples were obtained from healthy individuals and used as negative controls, while the remaining samples corresponded to patients at different stages of pancreatic cancer. Preliminary analyses were conducted by Ribeirão Preto Medicine School using the ELISA technique to quantify CA19-9 levels in each sample, providing reference values for comparison. Then, analyses of these samples were performed using the PDDA/PEDOT:PSS biosensor. For this, 10 μL of anti-CA19-9 was placed on the delimited interdigital surface by drop casting for 1 h and then washed with Milli-Q water. After the capacitance spectrum analysis was performed, 10 μL of the blood sample was immobilized for 10 min on the surface, washed with Milli-Q water, and then analyzed in the Solartron. The agreement between the two methods was assessed using the Bland-Altman plot, and a complementary analysis of the sensor response was conducted through IDMAP to explore clustering and classification patterns. All procedures involving human samples were approved by the institutional ethics committee (according to CONEP number 6.209.254), and informed consent was obtained in accordance with the Declaration of Helsinki.

### 2.8 Data Analysis

To analyze the high-dimensional data generated by EIS, particularly capacitance versus frequency measurements, dimensionality reduction techniques have become increasingly important in the field of biosensors.^7^ Linear projection techniques create new low-dimensional representations by calculating linear combinations of the original data attributes and arranging them on an orthogonal basis. A well-known example of this approach is Principal Component Analysis (PCA). In this context, multidimensional projection methods such as IDMAP provide a visual means of interpreting complex data sets by mapping them into two-dimensional spaces while preserving the similarity relationships between samples. IDMAP, implemented in the open-source PEx-Sensor’s software, applies a nonlinear projection strategy. In this approach, each data instance is represented as a spectrum *X* = {*x*1, *x*2,..., *xn*}, forming a set of *m*-dimensional data points. A dissimilarity measure δ­(*xi*, *xj*) quantifies the distance between two instances *i* and *j*. The projection generates a corresponding set *Y* = {*y*1, *y*2,..., *yn*} points in a lower-dimensional space, commonly two or three dimensions, where the Euclidean distance *d*(*yi*, *yj*) a (Euclidean) represents their proximity in the projection. More formally, a multidimensional projection is an injective function *f*: *X* → *Y* that attempts to minimize the discrepancy between the original and projected distances, aiming to make |δ­(*xi*, *xj*) – *d*(*f*(*xi*), *f*(*xj*))| as close to zero as possible for all pairs of data points *xi*, *xj* ∈ *X* (eq 5):^15^

$$S_{\mathrm{IDMAP}}=\frac{\delta (x_{i},x_{j})-\delta_{\min}}{\delta_{\max}-\delta_{\min}}-d(yi,yj)$$ 5

where *S*_IDMAP_ is the projection, δ_max_ e δ_min_ are the maximum and minimum Euclidean distances.

A key advantage of these modern techniques is their ability to combine scalability and accuracy. They efficiently process large data sets and generate interactive projections with high precision. The resulting two-dimensional maps approximate both global data structure and local relationships, where each point, displayed as a circle or node, represents a data element whose proximity reflects its similarity to others.^16^

## 3 Results and Discussion

### 3.1 Film Structure Analysis

PDDA/PEDOT:PSS thin films, fabricated using the layer-by-layer technique, were used to functionalize gold interdigitated electrodes. Decher and colleagues^17,18^ were among the pioneers in demonstrating that oppositely charged materials can be assembled into highly controlled thin films. In this way, even an extensive multilayer architecture can be easily prepared, since ionic interactions act as the driving force for film growth. Figure S1 (Supporting Information) shows the capacitance spectra of PDDA/PEDOT:PSS multilayers with 2, 4, 8, and 10 bilayers, as well, the electrode without film. Capacitance increases with the number of bilayers, mainly in the electrical double layer region disturbance. This result is consistent with the increased film thickness and enhanced charge-storage capability characteristic of multilayer architecture. Therefore, ten bilayers were selected as the optimal configuration, balancing high signal intensity, clear spectral distinction, and practical fabrication feasibility. Previous studies on PDDA/PEDOT:PSS LBL assemblies reported that both the film thickness and the UV–Vis absorbance increase systematically with the number of bilayers deposited, elucidating that absorbance increase is directly associated with a higher amount of PEDOT incorporated into the multilayer structure.^10^ PM-IRRAS operates by measuring the absorption of infrared light by molecular bonds and employs polarization modulation to selectively detect vibrational transitions oriented perpendicular to the reflective surface, providing high sensitivity for characterizing ultrathin films. Additionally, this technique enabled the direct assessment of the film structure on the electrode surface.^19,20^ Figure 1 shows the PM-IRRAS spectra of PDDA, PEDOT: PSS, and the 10 bilayers of the PDDA/PEDOT:PSS film. The PDDA spectrum exhibits characteristic peaks, including the C–H bending vibration of methylene groups (−CH_2_−) at 1450 cm^–1^, the C–N stretching of quaternary amines at 1190 cm^–1^, and a band at 966 cm^–1^ attributed to the stretching vibration of the =(CH_2_)­N^+^(CH_3_)_2_(CH_2_)=. Additional peaks at 825 and 866 cm^–1^ correspond to out-of-plane bending of aromatic ring hydrogen atoms, and the absorption band at 1636 cm^–1^ is attributed to O–H bending vibrations from water molecules.^21,22^ PEDOT:PSS also exhibited typical bands in the spectrum, with the symmetric stretching of the EDOT ring appearing at 1420 cm^–1^.^22–24^ The C–O–C stretching vibration of the EDOT ring is evident at 1066 cm^–1^, while the C–S stretching appears at 820 and 857 cm^–1^. The C=C band at 1534 cm^–1^ is related to the vibrations of the bonds involved in the bipolarons and, consequently, to the conductivity of the material.^25,26^ In the spectrum of the 10-bilayer PDDA/PEDOT:PSS film, a prominent peak is observed at 1632 cm^–1^, consistent with C=C stretching from both the phenyl side group and quinoid EDOT units. The spectrum of the self-assembled film confirms the presence of individual PEDOT:PSS and PDDA layers adsorbed on the electrode surfaces. In fact, this immobilized layer on the electrode capacitors is essential for maintaining the activity of the active layer, which justifies its use in biosensors.^22,27^

**Figure 1:**
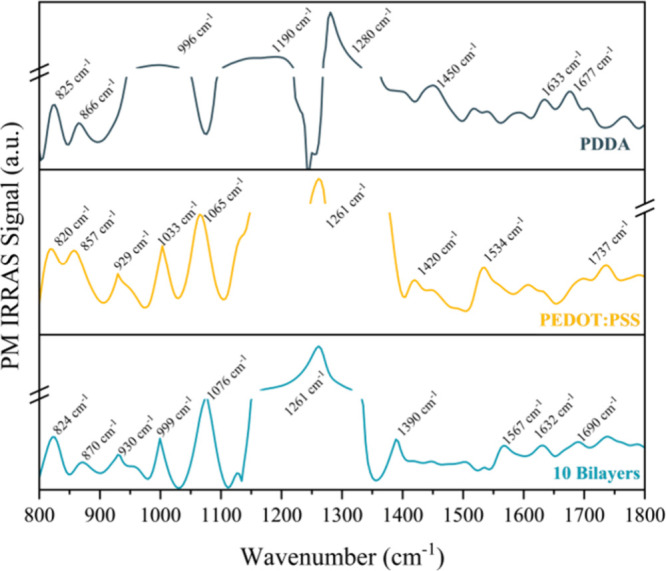
PM-IRRAS spectra of PDDA, PEDOT:PSS, and the film composed of 10 bilayers of PDDA/PEDOT:PSS sensors. Source: Author’s own.

### 3.2 Adsorption Kinetics

Adsorption kinetics were assessed based on capacitance (*F*) measurements at a frequency of 1 Hz. The kinetic analysis focused on 37 and 100 U/mL, as these concentrations are clinically relevant: 37 U/mL is the diagnostic threshold for pancreatic cancer, while 100 U/mL represents a high biomarker level. To minimize the effects of the matrix, the kinetics were plotted by Δcapacitance (*F*_AB_ – *F*_AG_). The experimental data were fitted to pseudo-first-order (PFO), pseudo-second-order (PSO), and Elovich kinetic models, as illustrated in Figure 2. A summary of the fitting results and the corresponding kinetic parameters is provided in Table 1.

**Figure 2:**
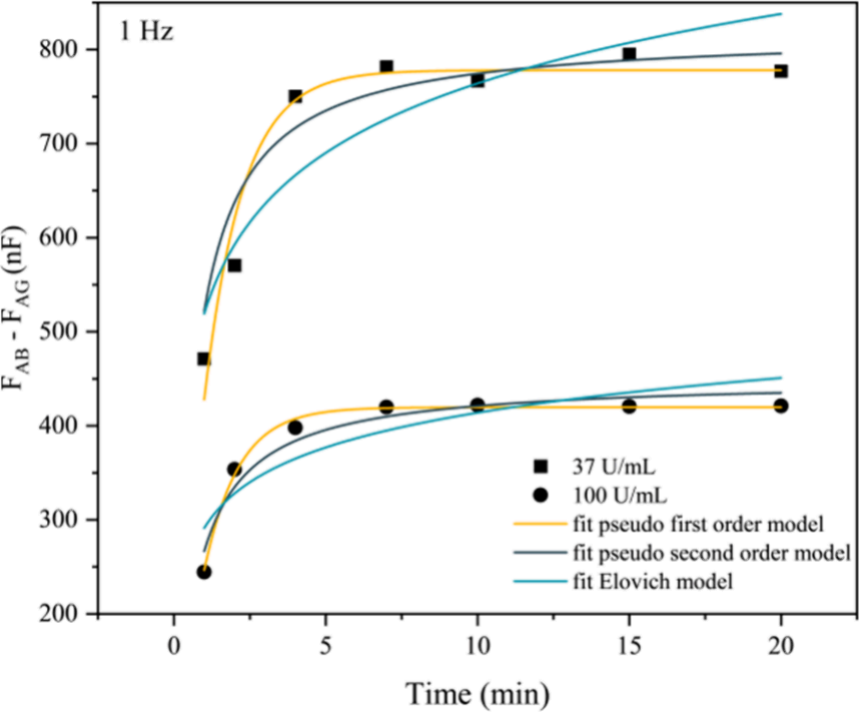
Adsorption kinetics of CA19-9 at a frequency of 1 Hz for concentrations of 37 and 100 U/mL as a function of time for the PDDA/PEDOT:PSS sensors. Source: Author’s own.

**Table 1:** Parameters of the Adjustments of the Kinetic Models^a^

pseudo-first-order model			pseudo-second-order model			Elovich model		
	37 U/mL	100 U/mL		37 U/mL	100 U/mL		37 U/mL	100 U/mL
*q*_e_	778.03 ± 14.85	419.63 ± 2.44	*q*_e_	818.41 ± 1.07	450.01 ± 11.05	α	13740.24 ± 16220.59	12496.62 ± 18833.61
*k*_1_	0.80 ± 0.07	0.89 ± 0.03	*k*_2_	0.002 ± 0.00004	0.003 ± 5.7 × 10^–4^	β	0.009 ± 0.002	0.019 ± 0.004
*R* ^2^	0.95	0.99	*R* ^2^	0.88	0.95	*R* ^2^	0.83	0.78

a Source: Author’s own.

The plots of Table 1 and Figure 2 indicate a rapid progression of the adsorption process, reaching equilibrium of the signal at 7 min regardless of the CA19-9 antigen concentration evaluated. Therefore, 10 min adsorption time was adopted for all subsequent experiments in this study. The adsorption curves showed the best fit to the PFO kinetic model (yellow curve). Initially proposed by Lagergren, this model assumes that the adsorption rate is proportional to the difference between the maximum adsorption capacity at equilibrium and the amount adsorbed at a given time. This behavior suggests a relatively fast initial adsorption phase, followed by a slowdown as the active sites on the adsorbent surface become occupied.^28–30^

### 3.3 Calibration Curve

EIS was employed to construct calibration curves for IDEs functionalized with CA19-9 antibodies/antigens, ranging from 10^6^ to 1 Hz. EIS evaluates the system’s electrical response across a range of frequencies when an alternating electric field is applied. When a biomarker, such as an antigen, interacts with the surface, functionalized with antibodies or specific recognition elements, physical phenomena can occur: disturbances in the electrical double layer (EDL) generating electrical modifications at low frequencies (1–10^3^ Hz), and alterations of the capacitor surface, generating changes in the spectra at intermediate frequencies (10^3^–10^4^ Hz).^31^ These perturbations on the EDL, especially in the Helmholtz layer, modify the spatial distribution of interfacial charges, affecting critical electrical parameters, such as capacitance and impedance.^32,33^ Figure S2 (Supporting Information) illustrates the relationship between capacitance and AG CA19-9 concentrations across different frequencies. Noted at higher frequencies, no significant capacitance differences were observed among the concentrations tested, once the electrical response in this domain is predominantly influenced by the substrate and variations in the nanostructured films. In contrast, measurements below 1 kHz emphasize the influence of the EDL, where specific variations resulting from AB/AG adsorption are most pronounced. In this regime, the capacitive response is directly governed by the dielectric properties and the adequate thickness of the adsorbed layer.^32,34^ A decreasing trend in capacitance is observed as the CA19-9 antigen concentration increases from 0.01 to 300 U/mL. This suggests that antigen–antibody binding reduces the charge density and redistributes them at the electrode–solution interface. Glycoproteins, such as CA19-9, have a dielectric constant significantly lower than that of water.^35^ Consequently, the displacement of interfacial water by adsorbed proteins can lower the effective dielectric constant, reducing capacitance. In addition, antigen binding may restrict ion mobility and hinder the reorganization of the EDL, as the biomolecule acts as an additional dielectric barrier that limits the accumulation of mobile charges near the electrode surface.^32,33^ To minimize intrinsic variations of the sensor, such as surface roughness, minor differences in the effective electrode area, film deposition imperfections, and instrumental or environmental fluctuations (e.g., temperature and humidity),^32^ the CA19-9 antigen concentrations versus frequency curves were normalized. The calibration curve was generated from normalized capacitance measurements taken at a frequency of 1 Hz (Figure 3). Data fitting was performed using the Langmuir–Freundlich isotherm model, which is suitable for systems characterized by heterogeneous binding site distributions.^29,36^ The results show a consistent increase in normalized capacitance F* with increasing CA19-9 concentration, confirming the sensor’s responsiveness to AG binding. High coefficients of determination (*R* ^2^ = 0.987 and 0.999) indicate excellent agreement between the model and the experimental data.

**Figure 3:**
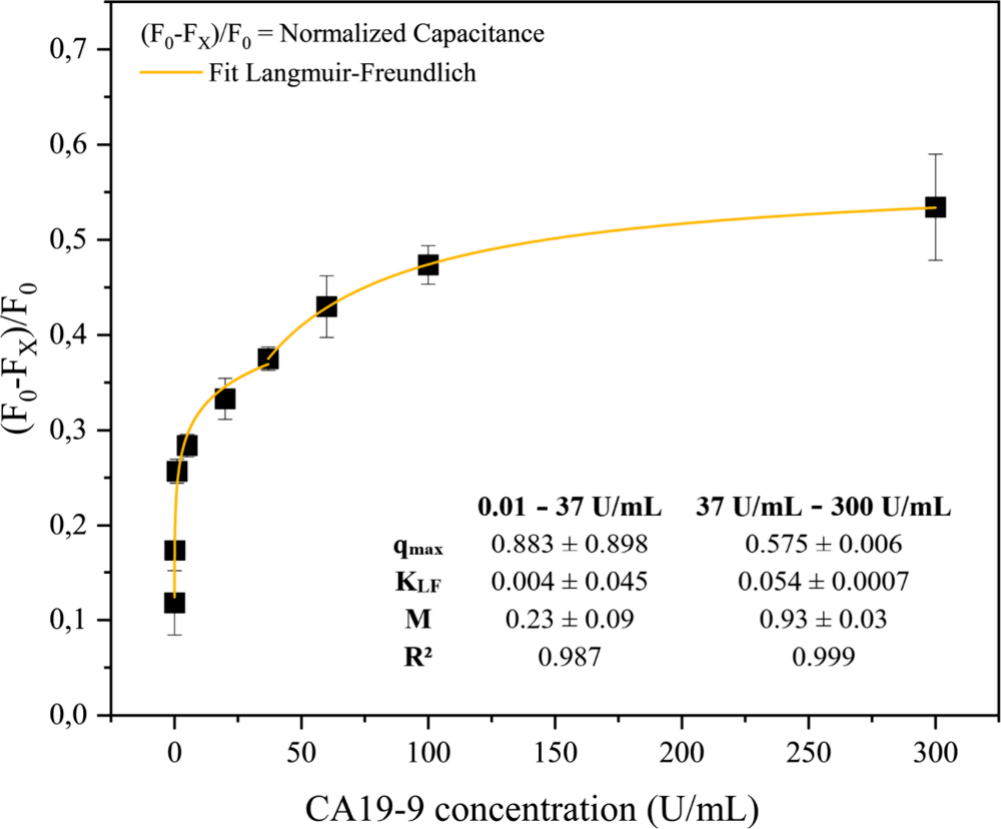
Normalized capacitance as a function of CA19-9 antigen concentration. Source: Author’s own.

The heterogeneity coefficient M in the Langmuir–Freundlich model reflects the uniformity of the binding sites on the sensor surface, ranging from 0 to 1. Values approaching 1 are indicative of more homogeneous surfaces.^36–38^ For a homogeneous system, all binding sites are identical in terms of binding energy and affinity for the adsorbate. In contrast, a heterogeneous system comprises binding sites with varying binding energies.^39^ For the PDDA/PEDOT:PSS biosensor, the system exhibits heterogeneous character up to 37 U/mL (*M* = 0.23 ± 0.09), while at concentrations above 37 U/mL, it becomes more homogeneous (*M* = 0.93 ± 0.03). Eqs 6 and 7 were derived based on the parameters obtained by fitting the model to the concentration ranges of 0.01–37 and 37–300 U/mL, respectively.

$$\mathrm{Concentration}\;(U/\mathrm{mL})=\frac{1}{0.004}{\left(\frac{y}{0.883-y}\right)}^{0.23}$$ 6

$$\mathrm{Concentration}\;(U/\mathrm{mL})=\frac{1}{0.054}{\left(\frac{y}{0.575-y}\right)}^{0.93}$$ 7

The IDMAP technique enables the visualization of the capacitance spectrum for different concentrations of AG CA19-9, by projecting capacitance spectra into points, reducing data dimensionality (Figure 4). In this representation, the proximity of points indicates similar sensor responses, as observed for the 1 and 5 U/mL concentrations.^15,16^ Additionally, the gradual left-to-right distribution reflects how the sensor response progressively increases with higher CA19-9 antigen concentrations, suggesting a linear or predictable behavior of the biosensor.

**Figure 4:**
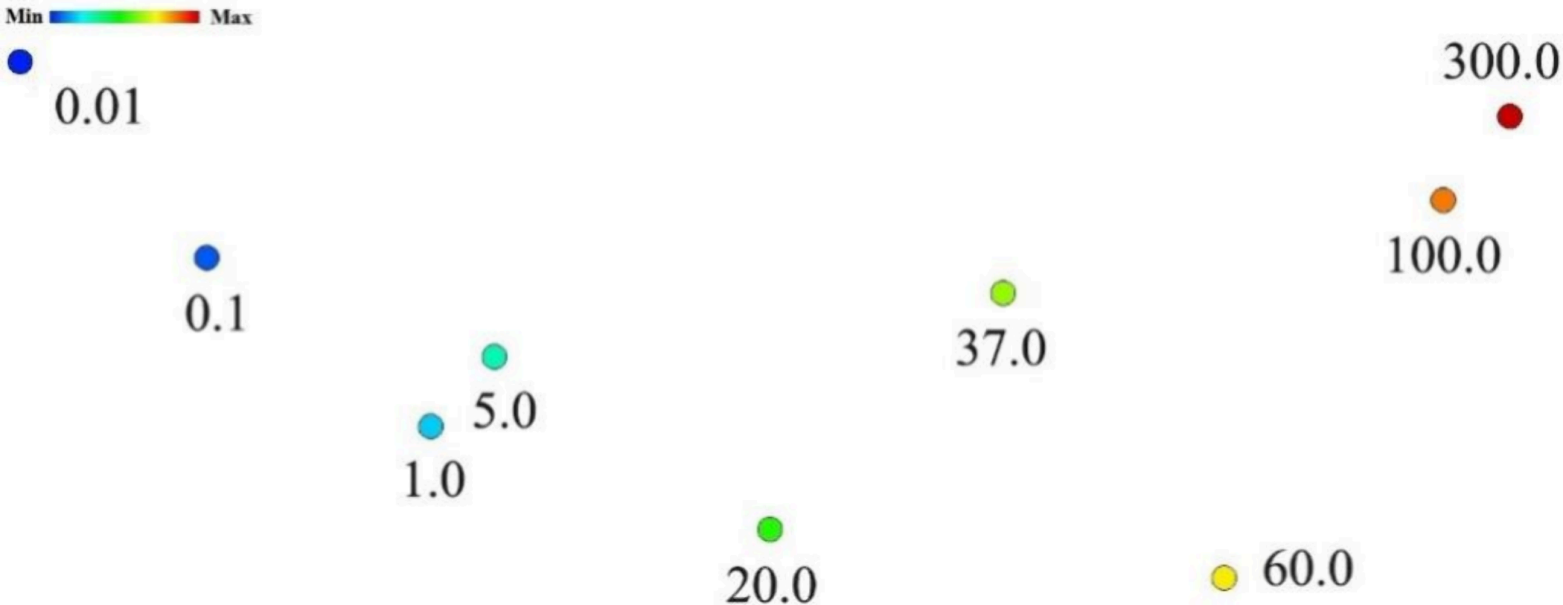
IDMAP of CA19-9 antigen concentrations for the PDDA/PEDOT:PSS biosensors. Source: Author’s own.

### 3.4 Sensibility

The noise standard deviation (σ_e_) was calculated by 10 measurements of the blank (AB), as illustrated in Figure S3 (Supporting Information). The standard deviation was 62.8 nF at 1 Hz, reflecting the intrinsic variability of the system in the absence of an analytical signal, i.e., the antigen. These values are critical for assessing the biosensor’s sensitivity, as they define the threshold below which minor signal variations cannot be distinguished from random fluctuations.^40^ Although this approach has limitations due to the nonlinearity of the calibration curve, which can lead to underestimation or overestimation of the LOD, the theoretical LOD was calculated according to IUPAC guidelines for comparison with literature reports. To mitigate the effects of nonlinearity, the Δcapacitance_(AB–AG)_ curve was analyzed on a logarithmic scale, as shown in Figure S4 (Supporting Information). Additionally, the limit of quantification (LOQ), defined as the lowest analyte concentration that can be quantified with acceptable accuracy and precision, was also determined.^13,14^ The LOD and LOQ values were determined to be 0.01 and 0.03 U/mL, respectively, indicating that the method can reliably detect low analyte concentrations. Indeed, these values are promising when compared to those reported in the literature. Soares et al.^41^ developed immunosensors based on electrospun nanofiber mats of polyamide 6 and poly­(allylamine hydrochloride), coated with either multiwalled carbon nanotubes (MWCNTs) or gold nanoparticles (AuNPs). The three-dimensional architecture enabled effective immobilization of CA19-9 antibodies for the pancreatic cancer biomarker, presenting a detection limit of 1.84 and 1.57 U·mL^–1^ for the nanostructured architectures containing MWCNTs and AuNPs, respectively. Huang et al.^42^ developed a label-free electrochemical immunoassay using polythionine–gold composites (AuNPs@PThi) for ultrasensitive and reliable detection of CA19-9, achieving a detection limit of 0.26 U/mL. Finally, Wang et al.^43^ produced a light-addressable photoelectrochemical biosensor by depositing uniform Bi_2_S_3_ films on ITO electrodes, modified with Au nanoparticles, and divided into discrete sensing zones. Antibodies for AFP, CEA, and CA19-9 were immobilized to enable multiplexed, label-free detection. For CA19-9, the sensor demonstrated a low detection limit of 0.01 U/mL, confirming its high sensitivity.

### 3.5 Selectivity

The biosensor response in the absence of immobilized antibodies (Figure 5a) was evaluated, and Figure 5b shows the corresponding IDMAP analysis for the PDDA/PEDOT:PSS sensor. The results demonstrate the effectiveness of a capacitance-based detection system for quantifying CA19-9 using electrodes functionalized with specific antibodies. The yellow curve, obtained without immobilized antibodies, served as a control and showed no significant variation in the capacitance with increasing AG CA19-9 concentration. The signal variation originates from nonspecific physicochemical interactions between CA19-9 and the PDDA/PEDOT:PSS multilayer, which is intrinsically sensitive to variations in charge distribution and dielectric properties at the electrode/electrolyte interface. In contrast, the blue curve, recorded with immobilized antibodies, displayed a clear concentration-dependent response, confirming specific antigen–antibody binding. IDMAP analysis further validated this distinction, with well-separated clusters corresponding to specific and nonspecific responses, supported by a high silhouette coefficient of 0.86. Some concentrations within the yellow cluster (e.g., 5, 20, 60 U/mL and 37, 100, 300 U/mL) were less distinctly separated, indicating limited differentiation among these concentrations without the AB immobilization.

**Figure 5:**
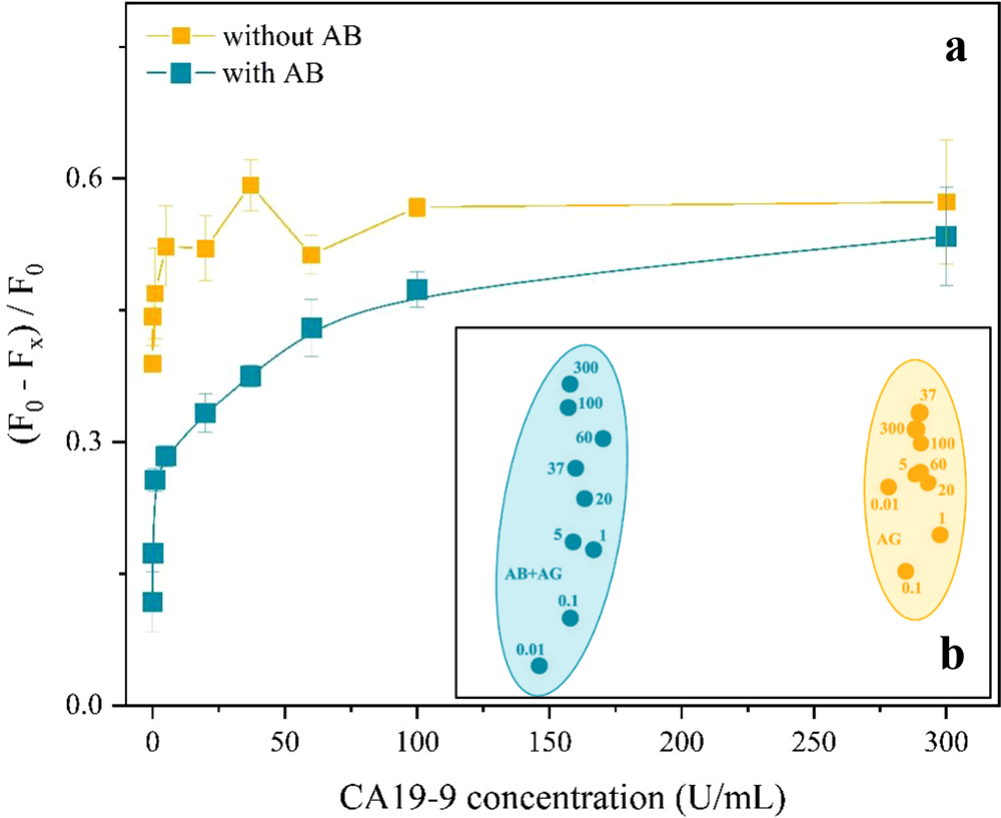
(a) Normalized capacitance with and without immobilized AB and (b) IDMAP of CA19-9 concentrations for PDDA/PEDOT:PSS-based biosensors. Source: Author’s own.

Figure 6a shows the plot of *F*_CA19‑9AB_ – *F*_AG_ for four specific biomarkers (P53, PSA, SARS-CoV-2, and CA19-9) as a function of frequency. At frequencies above 10 kHz, little difference is observed among the biomarker curves. However, at lower frequencies, the PDDA/PEDOT:PSS sensor exhibits distinct responses for each biomarker, with SARS-CoV-2 showing a marked decrease in *F*_CA19‑9AB_ – *F*_AG_. IDMAP analysis (Figure 6b) highlights the device’s selectivity for CA19-9, as indicated by the well-defined clustering of PSA, P53, and SARS-CoV-2. The silhouette coefficient of 0.69 further supports this clear separation, suggesting that the sensor can effectively discriminate among these biomolecules. Overall, the device demonstrates good performance for selective CA19-9 detection, although optimization may be needed to minimize interference from PSA. These findings highlight the sensor’s potential for clinical diagnostics and biomarker monitoring, providing a sensitive and selective approach for CA19-9 detection.

**Figure 6:**
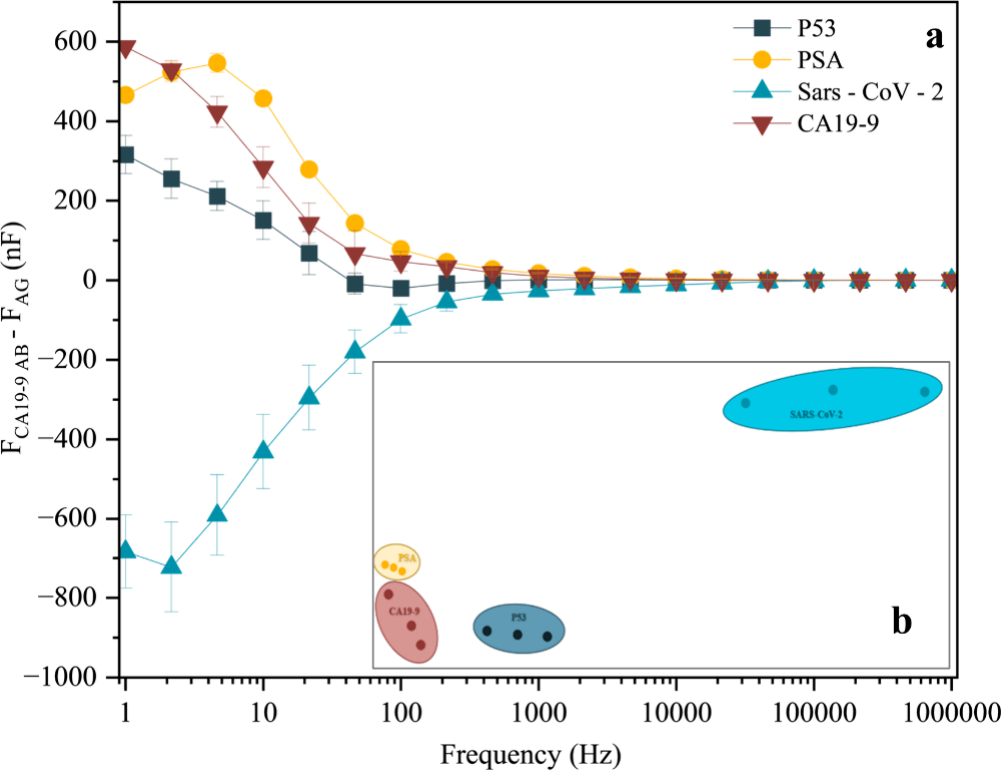
(a) *F*_CA19‑9AB_ – *F*_AG_ of biomarkers P53, PSA, SARS-CoV-2, and CA19-9 as a function of frequency; (b) IDMAP of biomarkers P53, PSA, SARS-CoV-2, and CA19-9 for PDDA/PEDOT:PSS-based biosensors. Source: Author’s own.

### 3.6 Detection Mechanism

The interaction between AB – AG is governed by molecular recognition specificity, playing a pivotal role in the selectivity and sensitivity of biomolecular sensors. This specificity minimizes interference from nontarget molecules, thereby enhancing the accuracy of the measurements. Nonspecific interactions can compromise sensor reliability, leading to false positives or negatives. A detailed understanding of the binding mechanism enables the development of strategies to optimize the analytical response of biosensors.^44–46^ Infrared spectroscopy has been widely employed for the structural characterization and analysis of proteins, particularly within the spectral region of 1700–1500 cm^–1^, where the characteristic amide I (1600–1700 cm^–1^) and amide II (1500–1600 cm^–1^) bands associated with peptide bonds are present. In this context, the AB–AG CA19-9 interaction was also investigated through these specific bands, given that CA19-9 is a glycoprotein, i.e., a carbohydrate chain linked to a protein backbone. Figure 7 presents the PM-IRRAS spectra of PDDA/PEDOT:PSS films for different concentrations of adsorbed AG CA19-9 in the 1550–1700 cm^–1^ range^41,47^ (the entire PM-IRRAS spectra are shown in the Supporting Information, Figure S5). The amide I (1665 cm^–1^) and amide II (∼1575 cm^–1^) bands exhibit intensity changes with increasing AG concentration, as shown in Table 2. The amide I band is predominantly associated with the stretching of the carbonyl group (C=O) from carboxylic acids in amino acids (80%), with minor contributions from C–N stretching (10%) and N–H bending vibrations (10%). The amide II band arises mainly from N–H bending (60%) and C–N stretching (40%) within the amide groups. In the analyzed spectra, the band at 1575 cm^–1^ showed an increase in area proportional to the CA19-9 antigen concentration, suggesting a higher density of amide groups in the film. Similarly, the band at 1665 cm^–1^ also increased with concentration, indicating intensified peptide interactions and supporting the hypothesis of CA19-9 adsorption onto the active sensing layer.^41,47,48^

**Figure 7:**
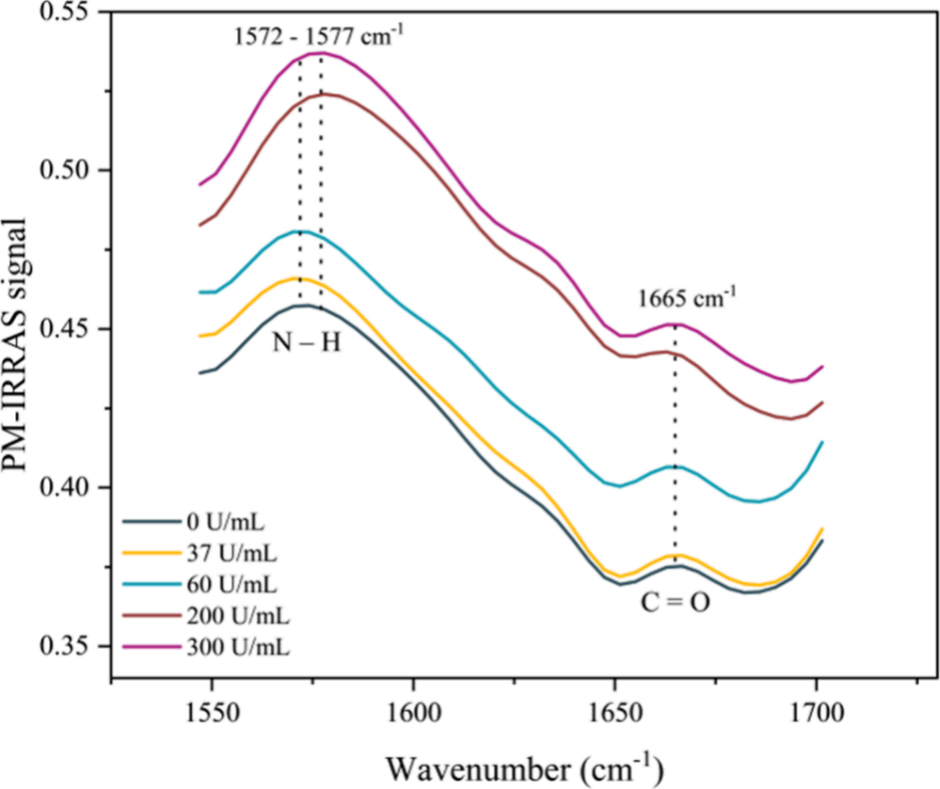
PM-IRRAS spectra of absorption bands ∼1575 and 1665 cm^–1^ for different concentrations of CA19-9. Source: Author’s own.

**Table 2:** Band Areas ∼1575 and 1665 cm^–1^ with the Adsorption of Commercial CA19-9 Antigens.

	band area (a.u.)	
CA19-9 antigen (U/mL)	**∼1575 cm^–1^**	**1665 cm^–1^**
0	44.21	11.47
37	44.86	12.99
60	46.73	13.95
200	52.96	18.42
300	54.03	18.86

AFM is a valuable tool for characterizing the morphology of molecular layers deposited on surfaces such as biosensors. Furthermore, this technique can provide direct evidence of AB–AG binding by enabling the visualization of topographical changes and confirming molecular interactions.^49^ The AFM images presented in Figure 8 show the topography of the PDDA/PEDOT:PSS-based biosensor at different stages of modification and CA19-9 biomarker adsorption. The root-mean-square (RMS) roughness (nm), summarized in Table 3, provided quantitative evidence of the AB–AG binding events. The PDDA/PEDOT:PSS film (10 bilayers) exhibited an RMS of 2.92 nm (Figure 8a), indicating a smooth and homogeneous surface, characteristic of a well-controlled and uniform film deposition. Following antibody functionalization, the RMS increased to 4.79 nm, accompanied by the appearance of small aggregates on the surface (Figure 8b). Upon adsorption of the CA19-9 biomarker at a concentration of 37 U/mL, significant topographical changes were observed, including a marked increase in RMS to 7.07 nm and the formation of larger aggregates, indicating specific AB–AG binding (Figure 8c). At a higher CA19-9 concentration (300 U/mL) (Figure 8d), the surface RMS further increased to 7.97 nm, although the increment was less pronounced. Additionally, the surface exhibited larger structures and a less uniform morphology.

**Figure 8:**
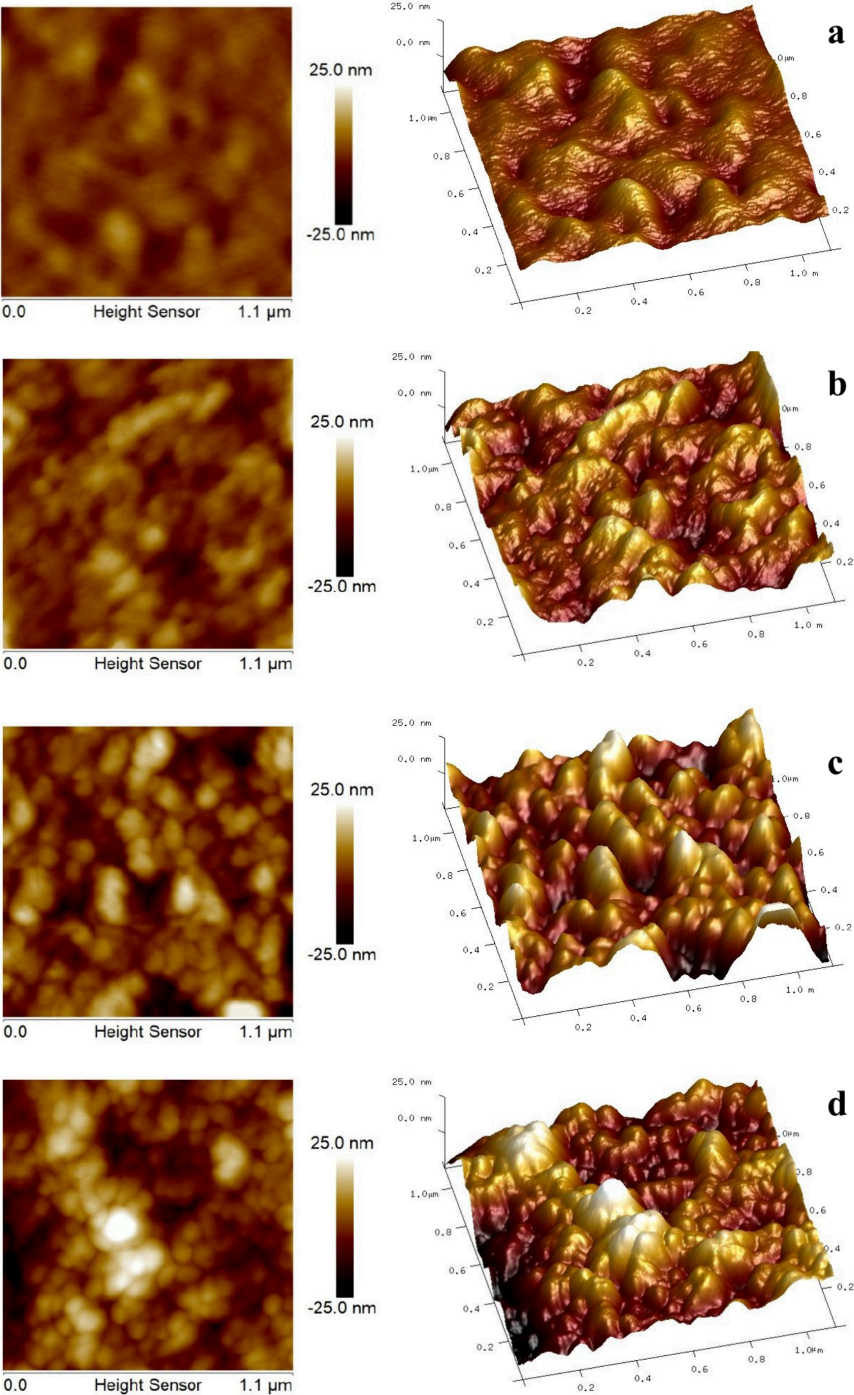
AFM topography of the PDDA/PEDOT:PSS biosensor: (a) film, (b) after functionalization with CA19-9 antibody, (c) following CA19-9 biomarker adsorption at 37 U/mL, and (d) after CA19-9 adsorption at 300 U/mL.

**Table 3:** Root Mean Square (RMS) Roughness (nm) of Biosensors Functionalized with Antibody (AB) and Antigen (AG)

	root mean square roughness
film	2.92 nm
AB	4.79 nm
AG 37 U/mL	7.07 nm
AG 300 U/mL	7.97 nm

### 3.7 Blood Sample Analysis

Comparative analysis was conducted on 24 blood samples from both healthy individuals and patients diagnosed with pancreatic cancer at different stages. Figure 9 presents a direct comparison between AG CA19-9 concentrations measured by the reference method ELISA (dark blue squares) and the PDDA/PEDOT:PSS-based biosensor (yellow circles). Visually, it is evident that ELISA measurements tend to yield higher values, particularly in samples with elevated biomarker concentrations. These divergences may arise from phenomena such as film saturation, recombination effects, or diffusion limitations of the antigen in blood. However, for healthy controls (the left region of the graph), both methods yield comparably low readings, indicating high sensor specificity. Notably, one false positive was identified in the control group (C3: 39.49 U/mL), while one sample from the diseased group (S496: 0.57 U/mL) presented as a near false negative. More measurements would be needed to understand the possible limitations of the biosensor. Although ELISA is the gold standard for antigen and antibody quantification, its accuracy can be compromised by interferences such as hemolysis, lipemia, and high metabolite levels. Additional challenges to measure the CA19-9 biomarker for both processes of evaluation include nonspecific interactions from heterophile or autoimmune antibodies, antibody instability, and environmental factors (pH, temperature, ionic strength) that affect binding efficiency and reproducibility, which could result in false positives and negatives.^50,51^ Besides, the presence of these false results may be due to factors such as sample collection error, older samples, identification errors, or an interferent in the blood. No correlation was identified between the positive and negative outliers with the patient’s health history, although the false negative was associated with a smoker and a diabetic patient. Outliers in these samples were analyzed at least five times,using different electrodes, confirming consistent results.

**Figure 9:**
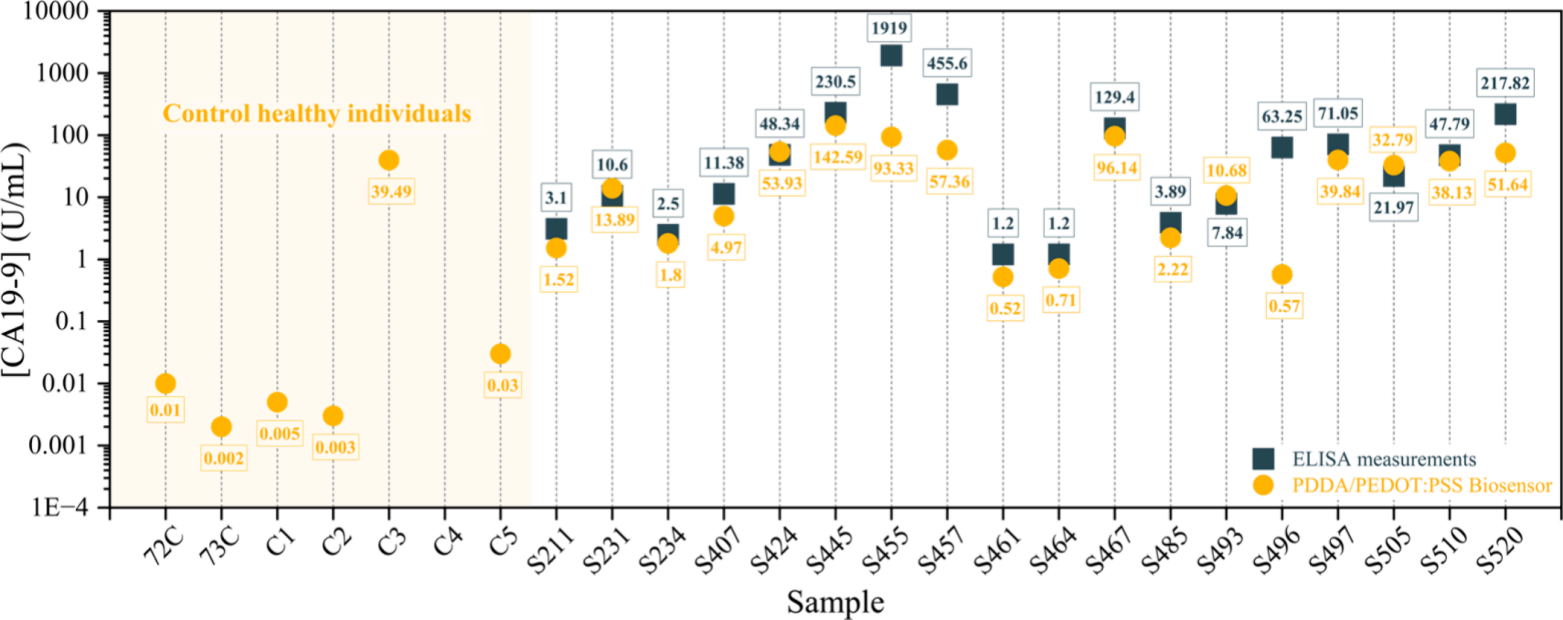
CA19-9 measurements of blood samples using ELISA and PDDA/PEDOT:PSS biosensor analysis. Source: Author’s own.

The performance of the biosensor was further evaluated using the Bland–Altman plot, as shown in Figure 10. This statistical tool is widely employed in analytical method validation to assess agreement between two distinct measurement techniques targeting the same analyte. Rather than correlating values directly, the Bland–Altman approach plots the difference between methods against their average, enabling detection of systematic bias, proportional error trends, and potential outliers.^52^ In this study, the Bland–Altman analysis was applied to compare CA19-9 concentrations measured by the PDDA/PEDOT:PSS biosensor with those obtained via ELISA, the gold standard reference technique. The plot presented all tested concentrations (Figure 10a) revealed a mean difference of approximately −102.57 U/mL, indicating a pronounced negative bias, i.e., the biosensor consistently underestimated CA19-9 levels in comparison to ELISA. Moreover, the limits of agreement (±1.96 SD) were wide (−811.63 to +606.49 U/mL), reflecting high variability and limited relative precision between the methods. This behavior aligns with observations for samples with elevated CA19-9 concentrations, particularly S445, S455, S457, S467, and S520, which showed substantial deviations from the ELISA results. After excluding higher concentration samples (CA19-9 > 100 U/mL), a second analysis was performed considering only concentrations below 100 U/mL (Figure 10b). The mean difference was reduced to −2.65 U/mL, indicating a substantial decrease in systematic bias. The new limits of agreement (−37.49 to +32.19 U/mL) were significantly narrower, suggesting improved agreement between the biosensor and ELISA under nonextreme clinical conditions. These findings indicate that the biosensor performs reliably at low to moderate CA19-9 concentrations; although its accuracy declines for samples with elevated biomarker levels, with a clear trend toward underestimation compared to ELISA.

**Figure 10:**
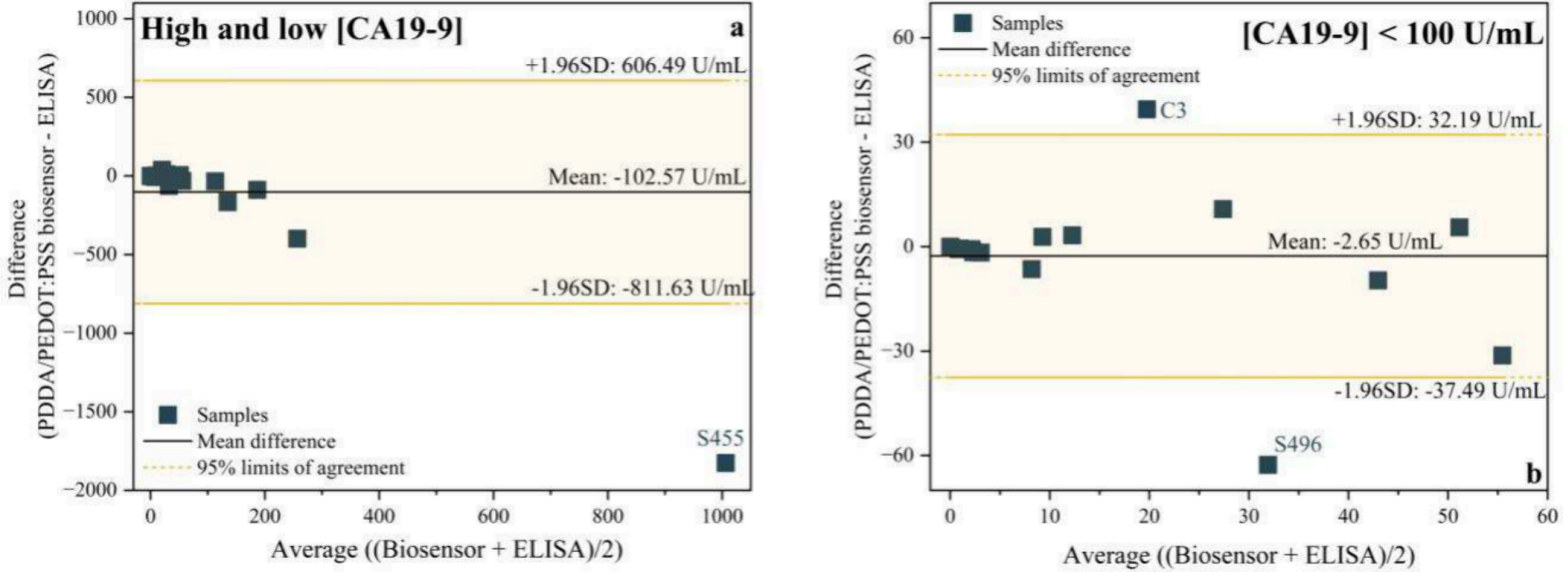
Bland–Altman analysis of ELISA and PDDA/PEDOT:PSS biosensor. (a) All concentrations of AG CA19-9 and (b) AG CA19-9 concentrations below 100 U/mL. Source: Author’s own.

Figure 11 presents the IDMAP projection for CA19-9 concentrations measured by the PDDA/PEDOT:PSS biosensor. Although a range of concentrations was obtained for individuals with pancreatic pathology, the diagnostic threshold of 37 U/mL, commonly used for pancreatic cancer detection, was adopted as the clinical reference. Consequently, the biosensor’s ability to differentiate samples above and below this threshold is a critical performance criterion. The IDMAP plot reveals three distinct regions: the lower-left quadrant (in yellow) corresponds to healthy control individuals; the upper-central region (light blue) comprises samples with CA19-9 levels below 37 U/mL; and the right-hand side (dark blue) clusters samples with more elevated CA19-9 concentrations (>37 U/mL). The dense clustering of samples below the 37 U/mL threshold, including all healthy controls, reflects a high degree of signal consistency and specificity within this subset, indicating the biosensor’s robust discriminatory capacity. Notably, sample C3 appears as a false positive, registering a biosensor response of 39.48 U/mL despite being a confirmed control (ELISA = 0). Its displacement from the control cluster within the IDMAP map confirms the sensor’s perception of this sample as dissimilar, highlighting the necessity of complementary validation strategies, such as cross-validation, multiplexed sensing, or data set training refinement to improve classification reliability.

**Figure 11:**
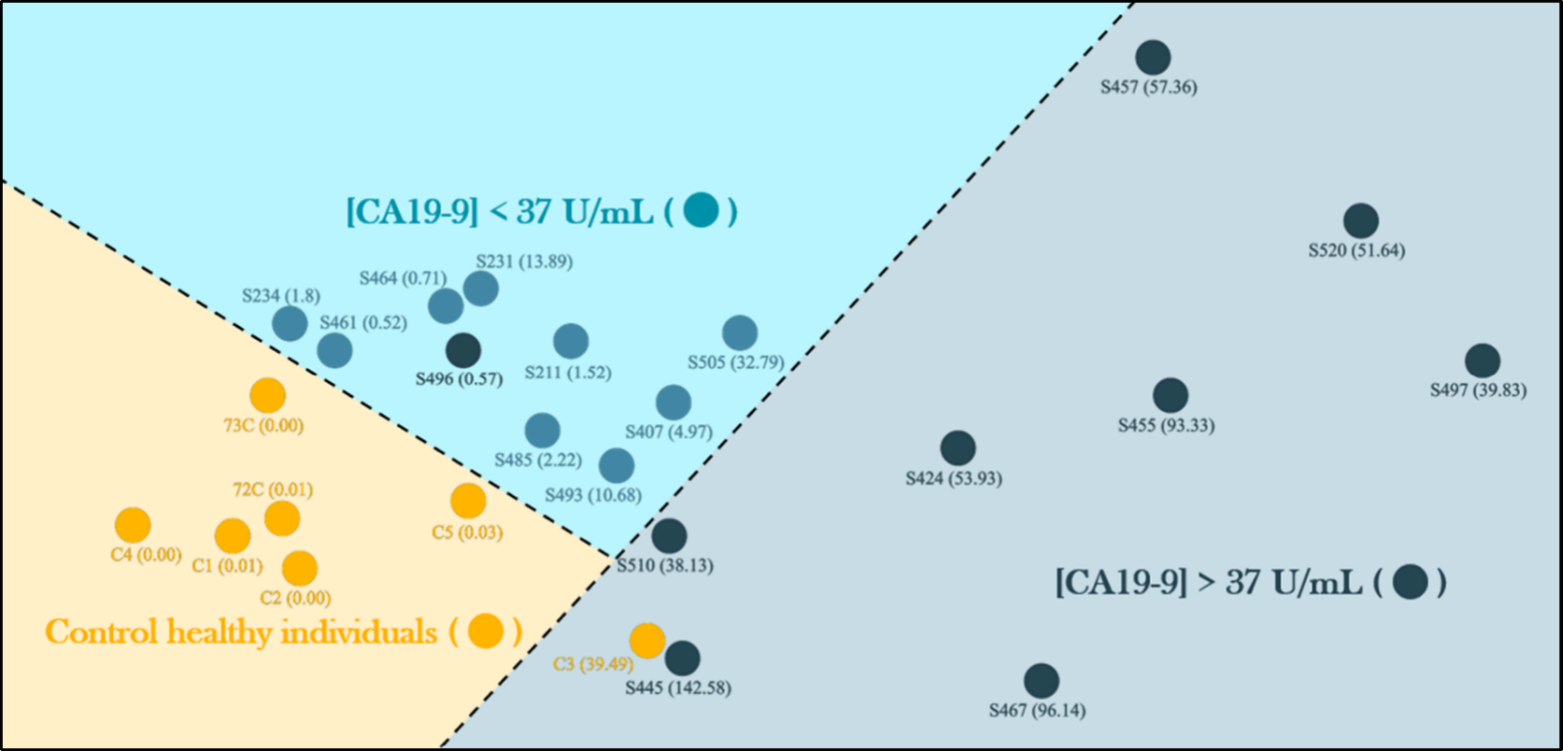
IDMAP of blood samples. Source: Author’s own.

On the other hand, the dark blue region reflects a pattern of dissimilarity among the samples, evidenced by the greater dispersion of data points. Although previous analyses indicate that the sensor tends to underestimate actual values at very high CA19-9 concentrations, the positioning of these samples on the IDMAP suggests that the device is still capable of recognizing internal similarity patterns within this subgroup. Notably, sample S496 (ELISA: 63 U/mL) exhibited distinct behavior compared to its group, as the sensor failed to reliably detect or differentiate it, potentially presenting a false negative. The IDMAP analysis demonstrates the system’s ability to discriminate between groups with reasonable structural fidelity, accurately identifying healthy individuals, distinguishing between low and intermediate concentrations, and clustering samples with similar high-response patterns. Despite limitations in absolute linearity, the IDMAP confirms that the sensor is capable of reliable classification, a key requirement for devices used in screening, monitoring, or multivariate classification algorithms. These findings suggest that the sensor holds strong potential for use in low-cost clinical screening applications.

## 4 Conclusions

This study demonstrates that multilayered PDDA/PEDOT:PSS biosensors represent a robust and versatile platform for the early detection of pancreatic cancer through selective recognition of the CA19-9 biomarker. The biosensors exhibited low detection and quantification limits, fitting well to the Langmuir–Freundlich model, and showed reliable discrimination of clinical samples at low-to-moderate CA19-9 concentrations. Importantly, analyses using patient blood samples confirm that the devices can approach the sensitivity of ELISA. Although deviations were observed at high antigen concentrations, likely associated with film saturation and matrix effects, the IDMAP analysis revealed that the system can still recognize internal similarity patterns, providing a path toward reliable classification. These results highlight not only the clinical potential of PDDA/PEDOT:PSS biosensors as low-cost alternatives for early stage cancer screening, but also their adaptability for integration with multiplexed platforms and machine learning algorithms. This study highlights the transformative role of biosensors as a foundation for future developments of diagnostic tools capable of overcoming one of the most urgent challenges in oncology.

## Supplementary Material



## Notes

Figure S1: capacitance versus frequency spectra of the gold interdigitated electrodes with different bilayers of PDDA/PEDOT:PSS; Figure S2: capacitance spectra of PDDA/PEDOT:PSS sensors with different concentrations of AG; Figure S3: capacitance spectra of the 10 blank measurements (AB); Figure S4: calibration curve on a logarithmic scale; Figure S5: PM-IRRAS full spectra for different concentrations of CA19-9 antigen (PDF)
