# Supplementary material for: Supramolecular PDDA/PEDOT:PSS Biosensor for Early Pancreatic Cancer Detection via CA19-9: Clinical Validation on Human Blood Samples
Source: ACS Omega. 2026 Jan 22;11(4):6452–63. doi: 10.1021/acsomega.5c11381 (PMC12878498; doi:10.1021/acsomega.5c11381)
Supplement: Supplementary file 1 [file ao5c11381_si_001.pdf]

Supramolecular PDDA/PEDOT:PSS biosensor for early pancreatic cancer detection via CA19-9: clinical validation on human blood samples

Gabriella Onila N. Soares[1*], Andrey C. Soares[2], Ronaldo Dias[3], Rafael Kemp[3], Débora Gonçalves[4]

[1] *University of São Paulo, Materials Engineering Department, Engineering School, São Carlos, São Paulo, 13563-120, Brazil. E-mail: gabriellaonila@usp.br.*

[2] *Federal University of Amazonas, Department of Physics, Manaus, Amazonas, 69067-005, Brazil.*

[3] *University of São Paulo, Ribeirão Preto Medical School, Ribeirão Preto, São Paulo, 14040-900, Brazil.*

[4] *University of São Paulo, Institute of Physics of São Carlos, São Paulo, 13566-590, Brazil.*

Supporting information

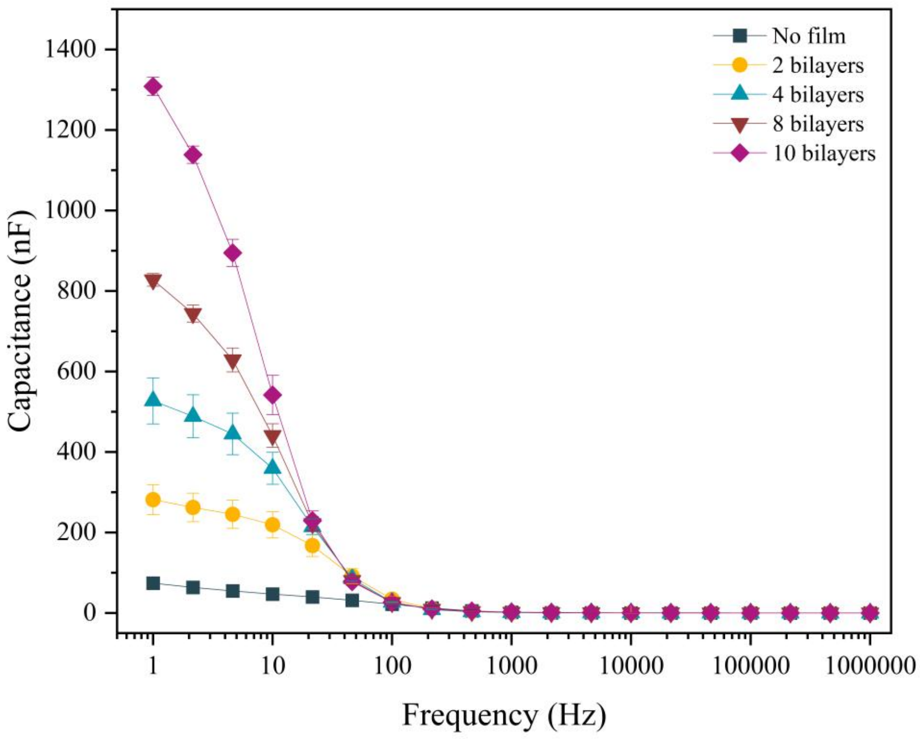

Figure S1: Capacitance versus Frequency spectra of the gold interdigitated electrodes with 0, 2, 4, 8, and 10 bilayers of PDDA/PEDOT:PSS. Source: Author's own.

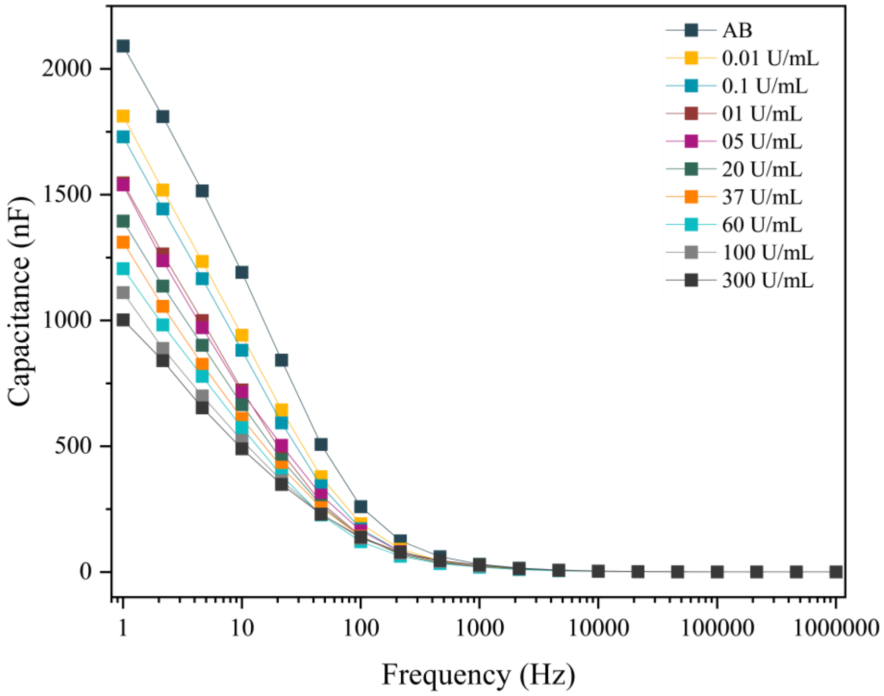

Figure S2. Capacitance spectra of PDDA/PEDOT:PSS sensors with different concentrations of AG. Source: Author’s own.

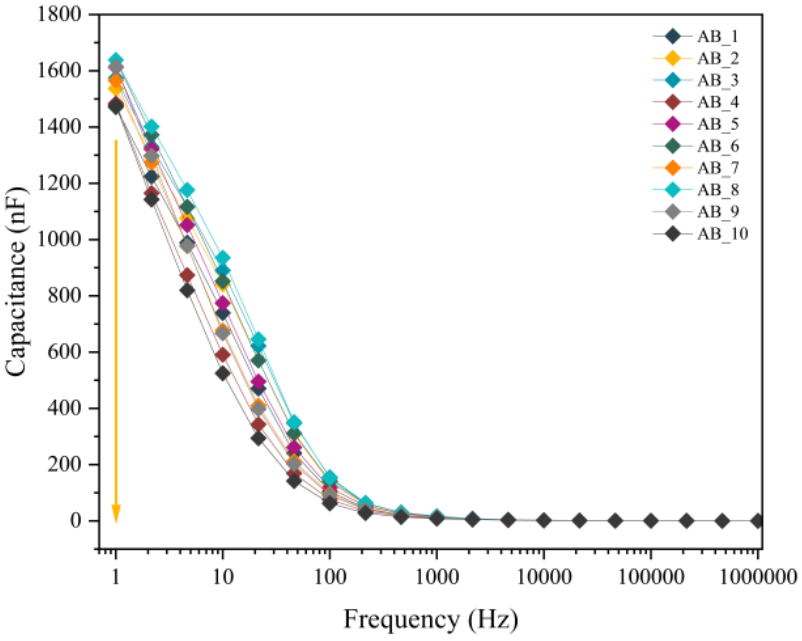

Figure S3. Capacitance spectra of the 10 blank measurements (AB) of the PDDA/PEDOT:PSS biosensor. Source: Author's own.

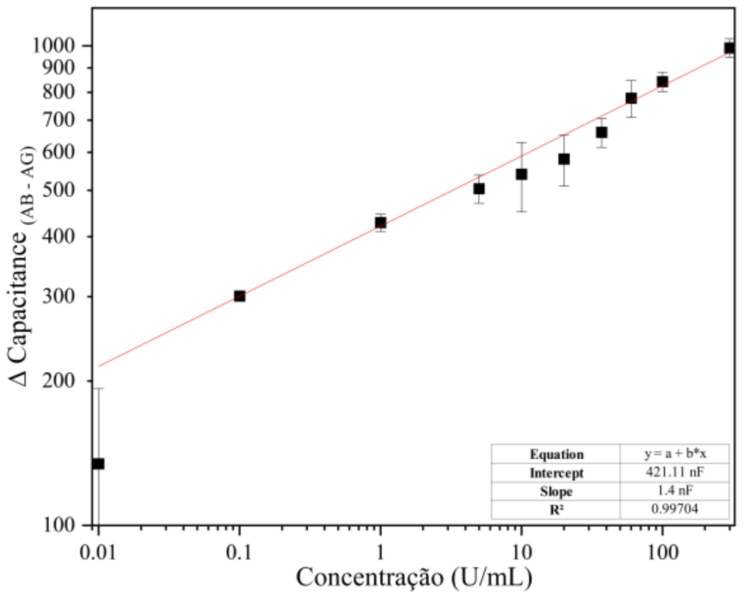

Figure S4. Calibration curve in logarithmic scale. Source: Author's own

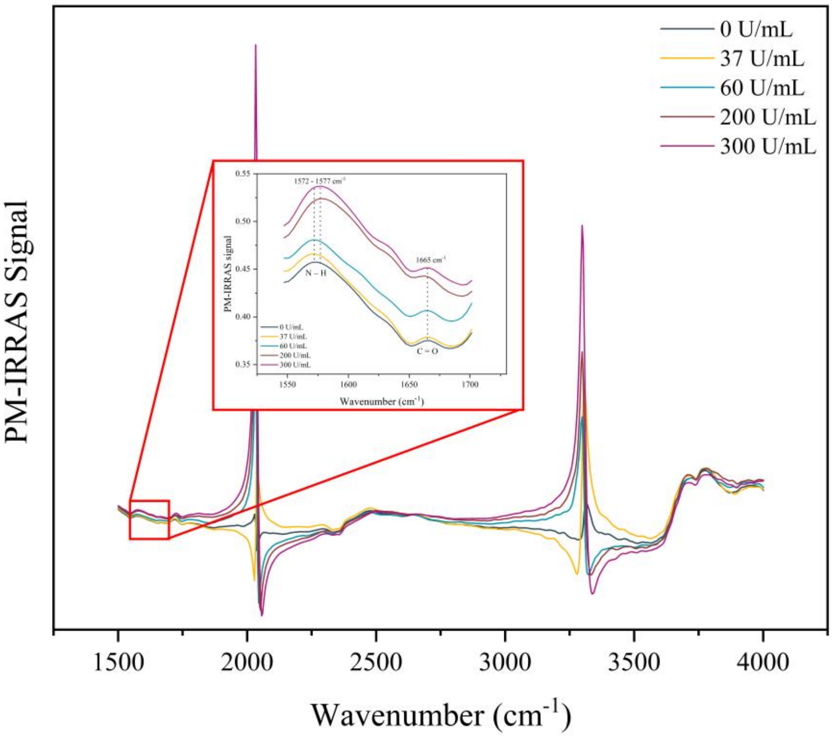

Figure S5: PM-IRRAS full spectra for different concentrations of CA19-9 antigen.
Source: Author’s own.
